# The Early Diagnosis and Treatment of Deformities of the Spine

**Published:** 1899-07-08

**Authors:** J. Jackson Clarke

**Affiliations:** Surgeon to Out-patients at the City Orthopædic and North-West London Hospitals


### HOSPITAL CLINICS AND MEDICAL PROGRESS.

### THE EARLY DIAGNOSIS AND TREATMENT OF DEFORMITIES OF THE SPINE.

Abstract of a paper read before the North-West District of the Metropolitan Counties Branch of the British Medical Association by J. JACKSON CLARKE, M.B.Lond., F.R.C.S., Surgeon to Out-patients at the City Orthopædic and North-West London Hospitals.

THE importance of recognising affections of the spine at the earliest possible time after their onset is so great that it is to me a matter of surprise that parents and those who have control of residential schools do not more frequently arrange for the periodical inspection of growing children by a medical man experienced in such work. If this were done more regularly it would not so often be left to the tailor or dressmaker to discover a deformity at a period when it is already far advanced, and probably no longer completely curable.

For the medical practitioner it is important not only to be able to recognise the character and degree of the deformity, but also to ascertain as fully as possible the pathological condition underlying it.

**The Chief Varieties of Deformity of the Spine.**—The terms used to designate the chief varieties of spinal deformity were introduced by Galen about the year 170 A.D. They are: Kyphosis, signifying a forward bowing of the spine, *e.g.*, that commonly termed "round shoulders"; Lordosis, signifying a forward convexity of the spine; and Scoliosis, generally used to signify a combination of lateral deviation with rotation of the spine.

**The Mode of Examining the Patient.**—The normal shape and range of movements of the different parts of the spine are to be remembered in making the examination. At birth the spine is nearly straight. The cervical curve convex forwards is developed by the child raising the head to look about it; the dorsal curve convex backwards is developed by the effect of the erector spinæ muscles when the child begins to sit up. The lumbar curve convex forwards only develops when the child begins to stand and walk. Only about the sixth or seventh year do these curves become permanent, *i.e.*, cease to disappear when the patient lies down. The normal range of movement in the spine is greater in the young than it is in the adult.

Little children, boys, and men should be entirely undressed; women and children should be undressed to the hips, so that the top of the intergluteal cleft is just visible; the garments can be fixed round the hips by a strap or safety-pin. The lower part of the skirts should also be pinned up so as to show the feet and ankles. A shawl may be thrown round the upper part of the body. A firm couch and a stool are required, in order that the patient may be examined when sitting and lying down as well as in the erect position. It is necessary to secure a good light.

**The Pathology of Spinal Deformity.**—It is to be regretted that certain deformities of the spine are commonly spoken of as though they were independent of any pathological origin. Thus one hears lateral curvature (scoliosis) frequently discussed, as though it were a pathological entity, whereas it is due to different causes in different cases. Thus it may be present at birth and depend upon developmental causes, it is not infrequently due to rickets, or to rheumatoid arthritis; in other cases the same deformity is secondary to a congenital wry-neck, and then may arise in the course of growth apart from any general pathological condition. The majority of cases of scoliosis arise in girls at the approach of puberty, and are attributed to "muscular weakness," "general debility," and "rapid growth." We should be on our guard against accepting such general observations as complete diagnoses; in such cases, by careful examination, a more definite opinion can be formed. Rickets is not uncommon about the age of puberty, and this affection is not limited to the poorer classes; anæmia and debility may be due to a great variety of causes, among which rheumatism and rheumatoid arthritis are to be remembered. Rheumatoid arthritis is no more restricted to adults than rickets is to the poor. Both rheumatoid arthritis and rickets are apt to recur in the same family; thus hereditary spinal deformities may be at the same time rachitic or osteo-arthritic. In certain families the children exhibit a rapid access of growth during certain years, and it is at such times that spinal deformity is apt to occur. Scoliosis again may be due to cicatricial shrinking of one lung, *e.g.*, after empyæma; to infantile paralysis; or again to hysteria. The same can be said for lordosis and for kyphosis. They, like scoliosis, are usually to be regarded as symptoms or effects rather than as definite pathological entities or diseases. None the less do they, like other serious symptoms, call for careful treatment. The terms "lateral curvature" and "angular curvature" are misleading. Instead of "angular curvature" it is better to use a descriptive term such as "tuberculosis of the spine." Not infrequently tuberculous disease shows itself by lateral curvature of the spine, and thus if the term "lateral curvature" is used as equivalent to a non-tuberculous curvature it will be seen what a confusion of ideas is involved. Before passing to consider the various deformities, I would recall the fact that one and the same pathological condition may be the predisposing cause of any of the three types of deformity of the spine. Thus rickets may lead the way to excurvation of the spine or kyphosis, to incurvation of the spine or lordosis, or, again, to scoliosis, which may be acompanied by dorsal kyphosis or dorsal lordosis, and by lumbar lordosis or by lumbar kyphosis. The determining causes of these various deformities are chiefly the *habitual postures* assumed by the patient in sitting, standing, and in sleep. We may take a school of 300 children and find more than half of them sit with the spine in a twisted position when they are doing writing exercises. Out of these 150, however, only one or two will be found to have scoliosis, and the reason that they have it is because their bones and ligaments are softer than they should be as much as from the fact that their muscles are weaker. It is impossible within the limits of the time at my disposal to discuss fully more than a few common deformities of the spine. I will, with your permission, show you typical instances of scoliosis and of tubercular and rheumatoid kyphosis.[1]

[1] Patients illustrating these conditions were shown at the meeting.

(*To be continued.*)

